# Usage of the Fungus *Mucor indicus* and the Bacterium *Rhodovulum adriaticum* in a Biorefinery System for Biochemical Production on Grass Hydrolysates

**DOI:** 10.3390/polym17030369

**Published:** 2025-01-29

**Authors:** Nenad Marđetko, Antonio Kolakušić, Antonija Trontel, Mario Novak, Mladen Pavlečić, Ana Dobrinčić, Vlatka Petravić Tominac, Božidar Šantek

**Affiliations:** University of Zagreb Faculty of Food Technology and Biotechnology, 10000 Zagreb, Croatia; akolakusic@gmail.com (A.K.); atrontel@pbf.hr (A.T.); mnovak@pbf.hr (M.N.); mpavlecic@pbf.hr (M.P.); adobrincic@pbf.hr (A.D.); vpetrav@pbf.hr (V.P.T.); bsantek@pbf.hr (B.Š.)

**Keywords:** biorefinery, grass hydrolysate, *Mucor indicus*, *Rhodovulum adriaticum*, biofuels, biochemicals

## Abstract

Utilization of various biomasses as raw materials in biorefineries represents a promising alternative for the production of valuable chemicals and biofuels. This study investigates the potential of the fungus *Mucor indicus* DSM 2158, cultivated on media containing the liquid phase of grass hydrolysates (LGH) and various nitrogen sources (yeast extract and corn steep liquor), for the production of valuable metabolites, such as ethanol, chitin, chitosan, and fatty acids. The ethanol yield varied depending on the cultivation media and conditions. The highest substrate-into-ethanol conversion coefficients (0.14–0.2 g g^−1^) were achieved during *M. indicus* cultivation on the LGH medium containing 5 g L^−1^ CSL in Erlenmeyer flasks and a bubble column bioreactor. In these cultivations, the highest fungal biomass concentrations (5.61–5.91 g L^−1^) were also observed. In flask cultivations, the highest content of total lipids in fungal dry biomass (15.76%) was observed. The obtained fungal biomass contained up to 22 fatty acids, with oleic acid (≈50%) being the most predominant. Chitin and chitosan yields were from 0.1 g g^−1^ to 0.3 g g^−1^ of dry biomass depending on the cultivation media and conditions. The residual media from the cultivation of *M. indicus* were used for the growth of the non-sulfur purple bacterium *Rhodovulum adriaticum* DSM 2781. Cultivations of *R. adriaticum* DSM 2781 on the residual media, in Erlenmeyer flasks and a stirred-tank bioreactor, resulted in a biomass yield of 0.50 to 2.26 g L^−1^. After extraction of bacterial biomass, total pigments (expressed as bacteriochlorophyll-a) were obtained in the range from 1.8 to 48.1 mg g^−1^ dry biomass depending on the media and cultivation conditions. The highest titer of bacteriochlorophyll-a was achieved during cultivation on the exhausted LGH medium with 5 g L^−1^ yeast extract. The established biorefinery system has to be optimized in order to reach capacity for transfer to a larger scale.

## 1. Introduction

Society today is under significant pressure to reduce its reliance on fossil fuels for energy production, as well as for the manufacture of various chemicals and materials. Due to uncertainties in securing adequate supplies of fossil raw materials such as oil, their adverse impact on the environment, and the rising cost of fossil fuels, it is essential to enlarge the share of alternative energy sources and promote the use of renewable raw materials [1,2]. This approach would help mitigate the negative effects of fossil fuel use and reduce humanity’s dependency on them.

Renewable biomass and its derivatives have emerged as promising alternatives for the production of these chemicals. The type of raw material has a crucial role in determining the method and extent of pretreatment required, which ultimately influences the cost-effectiveness, ecological footprint, and sustainability of the bioprocess [1]. In this context, lignocellulosic biomass, the most abundant biomass resource globally and a sustainable source of organic carbon, holds great significance [3].

On average, lignocellulosic raw materials consist of cellulose (30–50%), hemicellulose (20–40%), lignin (15–25%), and other components (5–35%) [4,5,6]. Unfortunately, substantial quantities of lignocellulosic biomass, often classified as waste, are incinerated despite its remarkable potential for transformation into high-value products, such as biofuels, biochemicals, and biopolymers [7].

Pretreatment of lignocellulosic raw materials is a crucial step in the production process. Various pretreatment methods exist, broadly categorized into physical, chemical, and biological approaches [8,9,10]. Following the hydrolysis of lignocellulosic materials, the resulting hydrolysate contains fermentable sugars, which serve as sustainable substrates for bioprocesses. These sugars can be utilized to produce valuable chemicals, such as ethanol, chitin, chitosan, pigments, sugar alcohols, and other high-value products [9].

Grass, as a renewable lignocellulosic feedstock, holds significant potential for use in biorefinery systems. It offers several advantages, such as a short growth cycle, moderate fertilization and irrigation needs, and a low lignin content, which reduces energy and chemical consumption during processing [11,12]. Furthermore, grass is not used in food production, thereby avoiding competition with food supply chains and preventing impacts on raw material prices in the food industry. Compared to sugar- and starch-based raw materials, grass is more economical but presents greater technological challenges, requiring suitable pretreatment methods to achieve high-value final products [13,14]. Currently, grass is primarily used as animal feed, but its industrial applications are growing. It is utilized in various bioprocesses, such as the production of biofuels (e.g., ethanol, hydrogen, and biogas) [15,16,17] and biochemicals (e.g., polyhydroxyalkanoates and organic acids) [18,19,20]. Additionally, grass is employed in co-combustion with coal or independent direct burning for heat and/or electricity production [21], paper production [22], lactic acid production on semi-solid substrates [23,24], amino acid production, and silage processing [25,26,27,28]. In silage production, 2.6 tons of grass silage is required to produce 1000 L of silage juice. From this, a total of 17.4 kg of amino acids can be extracted, including significant quantities of aspartate (1.47 kg), glutamate (1.24 kg), alanine (1.96 kg), and gamma-aminobutyric acid (1.46 kg) [28]. In 2023, the world market of amino acids was 1.4 million tons, with a further annual growth rate of 4.2% projected over the next few years [29]. Amino acids are widely used in the pharmaceutical and food industries.

For bioethanol production, grass offers several advantages, such as a favorable energy output/input ratio, availability, a low cost, and a higher ethanol yield. Ethanol production from grass can achieve yields ranging from 330 to 380 L per ton of dry grass matter [7,30,31]. In 2023, world fuel ethanol production was 113.297 billion liters [32]. For that amount of fuel ethanol (with an average yield of 350 L per ton of dry grass matter), 323.71 million tons of dry grass matter is needed.

For the manufacture of biofuels and other bioproducts, a variety of microorganisms and bioprocesses have been developed [10,14,18,33,34,35,36]. Fungi hold significant potential for application in biorefinery-type production, converting low-cost raw materials into high-value products [37]. Notable examples are fungi from the phylum *Mucoromycota* (commonly referred to as soil fungi), which thrive on simple sugar substrates and, in some cases, can assimilate more complex organic compounds [38,39]. 

*Mucoromycota* fungi are known for rapid growth and the ability to accumulate and secrete various metabolic products. This makes them promising candidates as powerful cell factories for application in a biorefinery. However, the use of fungi in biorefineries requires additional efforts to make it sustainable (economic and ecological) due to high resource and energy demands, waste generation, and expensive production processes. This situation is most obvious in biorefineries that produce only one product. To address this, bioprocess integration applying the biorefinery concept alongside the use of low-cost substrates can help optimize production, reducing both the time and costs associated with these bioprocesses.

*Mucor indicus* is a representative fungus from the phylum *Mucoromycota*. It is a non-pathogenic organism that forms white, fluffy colonies with small, dot-like sporangia. The cell wall of *M. indicus* is primarily composed of chitin and chitosan, which constitute up to 65.5% of its total cell wall composition. Other components include anionic polymers (such as polyphosphates and glucuronan), proteins, lipids, and minerals such as magnesium (Mg²⁺) and calcium (Ca²⁺) [38,39,40]. 

*M. indicus* can ferment different sugars into ethanol, including glucose, mannose, fructose, and galactose, with yields comparable to those achieved by the yeast *Saccharomyces cerevisiae*. Additionally, *M. indicus* can metabolize xylose, producing ethanol as its primary metabolic product [36,37]. During cultivation, *M. indicus* also accumulates significant amounts of intracellular lipids. Most of these lipids are polyunsaturated fatty acids, which have promising applications in food and biodiesel production [38,39].

Purple non-sulfur bacteria (PNSB) belong to the phototrophic purple bacteria group and represent its most diverse category [41,42,43,44]. A notable member of the PNSB group is *Rhodovulum adriaticum*, a Gram-negative, photosynthetic bacterium. Although PNSB can grow autotrophically using light and CO₂ as their sole carbon source, most species prefer photoheterotrophic growth in the presence of light and an external carbon source. These carbon sources can include organic acids (e.g., malate and succinate), alcohols, carbohydrates, fatty acids, and aromatic compounds [45,46,47]. 

PNSB are of significant interest from both ecological and economic perspectives. They can be employed in sustainable bioprocesses for various applications, such as hydrogen production as a biofuel [45,46], the synthesis of biochemicals like aminolevulinic acid [41,47], and the production of biopolymers such as polyhydroxybutyrate (PHB) [44,48]. PNSB also facilitate the synthesis of specific compounds, including carotenoids and photosynthetic pigments [49,50], as well as biomass production for various uses.

Additionally, PNSB play a crucial role in bioremediation and wastewater treatment. They effectively allocate nutrients from wastewater by assimilating soluble organics and nitrogen into biomass. This dual function of nutrient conversion and biomass generation makes PNSB-based wastewater treatment a highly attractive alternative to traditional methods, both economically and environmentally [51,52,53,54].

In our research, a two-stage integrated bioprocess system was developed utilizing *Mucor indicus* DSM 2185 and *Rhodovulum adriaticum* DSM 2781 for biorefinery-type production of various bioproducts.

In the first stage, *M. indicus* was cultivated on different media containing the liquid phase of grass hydrolysates (LGH) as the carbon source, supplemented with various nitrogen sources, such as a corn steep liquor (CSL) and yeast extract, to enhance bioprocess performance. The fermentation process resulted in the production of bioethanol and xylitol. Additionally, the fungal biomass was a rich source of valuable compounds, including biopolymers (chitin and chitosan) and fatty acids. After separating the biomass and bioethanol, the residual exhausted media were repurposed for the second stage of the integrated bioprocess.

In the second stage, *R. adriaticum* was cultivated under photoheterotrophic conditions using the exhausted LGH media. This process resulted in the production of isopropanol and an increased biomass yield, which was rich in pigments, specifically bacteriochlorophyll-a.

## 2. Materials and Methods

### 2.1. Experimental Setup

This study investigated the potential of selected microorganisms (*Mucor indicus* DSM 2185 and *Rhodovulum adriaticum* DSM 2781) for the production of various bioproducts. In the first stage, the effect of various carbon sources (glucose, xylose, and their combination) on the growth of *M. indicus* DSM 2185 was evaluated. Subsequently, *M. indicus* DSM 2185 was cultivated on media containing the LGH supplemented with varying concentrations of nitrogen sources, such as yeast extract (YE) and corn steep liquor (CSL). After the cultivation process, the fermented media were centrifuged, and the fungal biomass was harvested for the isolation and purification of chitin, chitosan, and microbial lipids. The residual supernatant, obtained by centrifugation, was then utilized in the second stage for the cultivation of *R. adriaticum* DSM 2781. The efficiency of the bioprocess was monitered by the appropriate analytical methods. Gravimetric methods were used to measure dry biomass, while total lipid contents were determined using extractive techniques. Spectrophotometric analyses were applied for quantication of pigment concentrations and optical density. Additionally, liquid chromatography (UPLC) was employed to assess carbohydrate and fermentation product levels, and gas chromatography (GC-FID) was used to analyze fatty acid composition of microbial lipids.

### 2.2. Feedstock, Media, and Working Microorganisms

In this research, dry mixed grass with a predominant hay content was utilized as a lignocellulosic raw material. The grass was shredded using a hammer mill (NA45; Megametal d.o.o., Kotoriba, Croatia) and sieved through a 5 mm mesh. Dilute acid pretreatment was performed in a high-pressure reactor at 180 °C with a residence time of 5 min, following the method described by Marđetko et al. [2].

Only the liquid phase of the grass hydrolysates (LGH) was used in this study. Its composition was determined using an ultra-performance liquid chromatography (UPLC) method (Section 2.5.2) and was expressed in g L^−1^ as follows: 2.21 ± 0.16 glucans, 7.31 ± 0.61 xylans, 3.34 ± 0.14 arabinans, 0.84 ± 0.09 acetic acid, and 2.47 ± 0.16 acid-soluble lignin. Unless otherwise stated, all chemicals used in this research were procured from Sigma-Aldrich (St. Louis, MO, USA).

*Mucor indicus* DSM 2185 and *Rhodovulum adriaticum* DSM 2781, obtained from the culture collection of the Laboratory for Biochemical Engineering, Industrial Microbiology, and Malting and Brewing Technology, Faculty of Food Technology and Biotechnology, University of Zagreb (Croatia), were used as the working microorganisms. 

The *M. indicus* DSM 2185 culture was maintained on standard potato dextrose agar (PDA; Difco, BD, Franklin Lakes, NJ, USA), and potato dextrose broth (PD broth; Difco, BD, Franklin Lakes, NJ, USA) was used for inoculum preparation. The media were sterilized in an autoclave at 121 °C for 15 min. Spores were collected from the PDA plates by adding a Tween 80 solution. After collection of spores, the number of spores in suspension was determined in a Thoma chamber. Depending on the number of spores in suspension, the exact volume (up to 5 mL) was defined so that 10^6^ spores were added (under aseptic conditions) to the flask containing 250 mL of nutrient medium for inoculum cultivation. The inoculum was cultivated on a shaker at 140 rpm at 30 °C for 48 h.

In the first stage of *M. indicus* DSM 2185 cultivation, media with varying carbon source concentrations were prepared in Erlenmeyer flasks. The media compositions (in g L^−1^) were as follows: 20 glucose, 10 xylose, and a combination of 20 glucose and 10 xylose. All media contained 5 yeast extract, 2 diammonium sulfate, and 2 diammonium hydrogen phosphate. The media (230 mL per flask, pH 5.5) were sterilized in an autoclave at 121 °C for 15 min. Cultivations were conducted under both aerobic and anaerobic conditions to evaluate the growth and metabolic performance of *M. indicus* DSM 2185. In aerobic cultivations, the flasks were shaken on a rotary shaker (200 rpm) and sealed with a cotton plug to permit air inflow and released-gas outflow. However, in anaerobic cultivations, the flasks were not shaken and were sealed with a fermentation airlock, allowing gases to exit but preventing the entry of fresh air.

In the second stage, cultivations of *M. indicus* DSM 2185 were conducted using the LGH. To detoxify the medium and remove substances inhibitory to growth and fermentation, the LGH was treated with activated charcoal before sterilization. Specifically, 25 g L^−1^ of activated charcoal was added to the LGH, and the mixture was shaken at 200 rpm at 30 °C for 1 h. The treated mixture was then filtered using a Büchner funnel and subsequently used for cultivation. The LGH media were supplemented with 1, 5, or 10 g L^−1^ of YE or CSL as a nitrogen source. The prepared media (230 mL per Erlenmeyer flask, pH 5.5) were sterilized in an autoclave at 121 °C for 15 min prior to inoculation with *M. indicus* DSM 2185.

*R. adriaticum* DSM 2781 cultures were maintained in a liquid medium that contained the following compounds (in g L^−1^): 2.7 malate, 2 sodium glutamate, 1.5 yeast extract, 0.8 (NH_4_)_2_SO_4_ × 7 H_2_O, 0.5 KH_2_PO_4_, 0.5 K_2_HPO_4_, 0.2 MgSO_4_ × 7 H_2_O, 0.053 CaCl_2_ × 2 H_2_O, 0.0012 MnSO_4_ × 7 H_2_O, 0.001 g each of nicotinic acid and thiamine chloride, and 10^−5^ g biotin. The pH of the medium was adjusted to 7.0 by adding a 4 M sodium hydroxide solution. The same medium was used for inoculum preparation. The medium was sterilized, and thermosensitive components were added after sterilization and cooling through a 0.2 μm pore-size filter (CHROMAFIL Xtra PA-20/25; Duren, Germany). Cultivations were conducted in 500 mL Erlenmeyer flasks, with a total nutrient medium volume of 250 mL. The inoculum was grown under microaerophilic conditions (due to the oxygen dissolving from remaining air in the flasks) on a shaker at 50 rpm, for 48 h at 30 °C, under 2000 lux warm white light, in flasks sealed with ground-glass stoppers.

The exhausted LGH media obtained after centrifugation of the *M. indicus* DSM 2185 cultures were used as media for the cultivation of *R. adriaticum* DSM 2781. After centrifugation, the supernatant was separated from the biomass by decantation. A volume of 225 mL of the medium was transferred to an Erlenmeyer flask and sterilized in an autoclave at 121 °C for 15 min. After cooling, 5 mL of a previously prepared solution of salts and vitamins (final concentrations, in g L^−1^: 0.053 CaCl_2_ × 2 H_2_O, 0.0012 MnSO_4_ × 7 H_2_O, 0.001 each of nicotinic acid and thiamine chloride, and 10^−5^ biotin) was added to the medium under aseptic conditions.

### 2.3. Microbial Cultivations in Erlenmeyer Flasks

Cultivations of *M. indicus* DSM 2185 were conducted in 500 mL Erlenmeyer flasks, with a total cultivation media volume of 250 mL. In the first stage, media with different carbon sources (glucose, xylose, and a mixture of glucose and xylose) concentrations (see Section 2.2) were inoculated with 20 mL of *M. indicus* DSM 2185 inoculum. In the second stage, LGH media with varying concentrations of nitrogen sources (see Section 2.2) were also inoculated with 20 mL of *M. indicus* DSM 2185 inoculum. In both stages, cultivations were carried out on a shaker at 200 rpm and 30 °C. The total duration of these cultivations was up to 72 h, with samples taken at predetermined periodic intervals for UPLC analysis. At the end of the second stage of the *M. inidicus* DSM 2185 cultivations, the contents of the flasks were centrifuged, and the biomass was dried and weighed to determine the final biomass concentration, as well as chitin, chitosan, and fatty acid contents.

For the cultivation of *R. adriaticum* DSM 2781, the exhausted LGH medium supplemented with a vitamin and salt solution was used. A volume of 230 mL of this medium was inoculated with 20 mL of *R. adriaticum* DSM 2781 inoculum. The cultivations were conducted in 500 mL Erlenmeyer flasks (sealed with ground-glass stoppers) under a warm white light intensity of 2000 lux, at 30 °C in microaerophilic conditions (due to the oxygen dissolving from remaining air in the flasks) on a shaker at 50 rpm. The cultivations lasted for 7 days, with samples taken every 24 h for UPLC analysis and optical density determination. At the end of cultivation, the contents of the flasks were centrifuged, and the biomass was dried and weighed to determine the final biomass concentration and for pigment isolation. All cultivations were performed at least in duplicate, and the data shown are the averages of these measurements.

### 2.4. Microbial Cultivations in the Bubble Column and Stirred-Tank Bioreactor

*M. indicus* DSM 2185 was cultivated in a bubble column bioreactor. Activated charcoal-treated LGH medium, supplemented with 5 g L^−1^ CSL, was used as the nutrient medium. Both the nutrient medium and the bioreactor were sterilized together by indirect steam at 121 °C for 15 min. The total volume of the nutrient medium was 1.5 L, with an inoculum volume of 8% (*v*/*v*). The cultivation was conducted with aeration at 2.0 L min^−1^ to ensure mixing of the broth in the bioreactor. The temperature was maintained at 30 °C, the pH of the medium was not adjusted during cultivation, and foam formation was controlled by adding Antifoam 204. Samples were taken at intervals over 48 h and analyzed using the UPLC method (see Section 2.5.2). At the end of the cultivation, the entire contents of the bioreactor were centrifuged, and the biomass was separated, dried and weighed to determine final biomass concentration as well as contents of chitin, chitosan, and fatty acids. 

The cultivation of *R. adriaticum* DSM 2781 was carried out in a stirred-tank bioreactor. After the cultivation of *M. indicus* in the bubble column bioreactor and the separation of the fungal biomass by centrifugation, 1 L of residual liquid medium was obtained. To this nutrient medium, 15 mL of a vitamin solution and 15 mL of a trace element solution were added, and the medium was inoculated with an 8% (v v^−1^) suspension of *R. adriaticum*. The cultivation lasted for 7 days at 30 °C, with a stirrer rotation of 50 rpm, under anaerobic conditions and warm white light. Samples were taken every 24 h for UPLC analysis and optical density determination. At the end of the cultivation, the entire bioreactor content was centrifuged, and the biomass was dried and weighed to determine the final biomass concentration and for pigment isolation. All cultivations were performed at least in duplicate, and the data presented here are the averages of these measurements.

### 2.5. Analytical Methods

#### 2.5.1. Isolation of Chitin and Chitosan

For the isolation of chitin and chitosan, 1 g of dried and ground biomass of *M. indicus* DSM 2185, obtained from various cultivation setups, was used. The biomass was suspended in 30 mL of 4 M sodium hydroxide solution and autoclaved for one hour at 121 °C. The alkaline-insoluble fraction was separated by centrifugation (6800 rpm for 15 min). The resulting supernatant was decanted, and the insoluble precipitate was washed to neutral pH (pH = 7) and then dried. To the dried and ground neutralized precipitate, 40 mL of 10% (v v^−1^) acetic acid solution was added, and the resulting suspension was refluxed and occasionally stirred for 6 h. Chitin was separated by centrifugation (6800 rpm for 15 min), washed with distilled water until neutral, and then dried. Chitosan was isolated from the supernatant by adding 4 M NaOH to raise the pH to 9. Chitosan was separated by vacuum filtration using a Büchner funnel. The chitin and chitosan crystals were washed with distilled water, ethanol, and acetone. The obtained crystals were dried at 60 °C until a constant weight was achieved [55,56].

#### 2.5.2. UPLC Analysis

Carbohydrate, alcohol, and organic acid composition during fermentation were determined by UPLC-RID (Ultra-Performance Liquid Chromatography–Refractive Index Detector) analysis, according to Marđetko et al. [2]. Samples withdrawn during cultivations were centrifuged to obtain the supernatant. The supernatant was then mixed in a 1:1 (v v^−1^) ratio with a 100 g L^−1^ ZnSO_4_ × 7H_2_O solution to precipitate proteins. The precipitated proteins were separated by centrifugation (10,000 rpm for 10 min). The supernatant was subsequently filtered into vials through a nylon syringe filter (CHROMAFIL Xtra PA-20/25; Duren, Germany) with a pore diameter of 0.2 μm. The prepared samples were analyzed by liquid chromatography (Agilent Technologies 1290 Infinity II; Santa Clara, CA, USA). A volume of 10 μL of each sample was injected into the UPLC system. Analytical column, Rezex ROA-Organic Acid H⁺ (15 cm × 7.2 mm; Phenomenex, Torrance, CA, USA), was used for the analysis. Elution was performed isocratically with 0.0025 M H_2_SO_4_ as the mobile phase at a flow rate of 0.6 mL min^−1^. The column oven temperature was set to 60 °C, and the temperature of the RI detector was set to 40 °C.

#### 2.5.3. Optical Density Determination and Gravimetric Analysis

Samples were thoroughly homogenized before analysis, and measurements were taken using 10 mm wide glass cuvettes (Hellma Optik GmbH, Jena, Germany). Biomass concentration was determined either by measuring the optical density at a wavelength of 660 nm using a Cary 100 UV-Vis spectrophotometer (Agilent Technologies, Santa Clara, CA, USA) or by determining the dry biomass weight [2].

#### 2.5.4. GC-FID Analysis

The composition and concentration of fatty acids in the *M. indicus* DSM 2185 biomass were determined using gas chromatography with flame ionization detection (GC-FID). Prior to the GC-FID analysis, the fatty acids in the samples were transesterified into methyl esters, followed by extraction into the appropriate solvent, in this case, hexane.

The transesterification of fatty acids into methyl esters was carried out following the NREL protocol [57]. A sample of 5 to 10 mg of *M. indicus* DSM 2185 biomass was weighed into 10 mL glass vials and dried in a desiccator overnight. Then, 20 µL of methyl tridecanoate (C13:0ME = 10 mg mL^−1^; internal standard 1), 200 µL of chloroform solution (2:1, vol vol^−1^), and 300 µL of 0.6 M HCl solution were added to the vial. The contents were mixed using a vortex mixer and then placed in a water bath at 85 °C for 1 h.

Afterwards, the vials were cooled to room temperature, and 1 mL of hexane was added. The contents of each vial were mixed again using a vortex mixer to extract the formed esters into the hexane phase, then left for 1 h at room temperature to allow phase separation. The upper hexane layer could have been diluted with additional hexane if necessary. A volume of 200 µL of the diluted hexane phase was pipetted into a gas chromatography vial, to which 5 µL of pentadecane (*γ* = 1 mg mL^−1^; internal standard 2) was added. The vial was then sealed with a PTFE/silicone cap. The prepared sample was used for the analysis of fatty acid esters by gas chromatography with flame ionization detection (GC-FID).

GC-FID analysis was performed on a GC-2010 Plus AF gas chromatograph (Shimadzu, Kyoto, Japan). The method conditions/parameters for the analysis of fatty acid ester composition are shown in Table 1. Identification and quantification of fatty acid esters were performed by comparing retention times with calibration charts derived from a standard mixture of 37 fatty acids (F.A.M.E. C4–C24; Supelco, Bellefonte, PA, USA). The concentration of fatty acids was determined by calculating the area under the peak using GC Solutions version 2.32 software and the integrator built into the device. The composition of methyl esters of fatty acids is expressed as the yield of each fatty acid per gram of dry biomass (mg g^−1^) and as the mass fraction (%) of total fatty acids in the sample.

The calculation of total fatty acids was performed according to the following formula:

(1) $${Total\;FAME}_{{C13}_{normal}}=\sum_{C4-C24}\frac{{FAME}_{Ci}}{{FAME}_{C13}}\times c_{added\;FAMEC13}$$

Total fatty acids were calculated as a percentage of the dry biomass (*m*) used for transesterification:

(2) $$\%\;total\;FAME=\frac{{total\;FAME\;C13}_{normal}}{m}\times 100$$

where FAME_ci_ is the amount of the measured FAME (fatty acid methyl ester), FAME_c13_ is the amount of the measured C13 standard, c_added FAMEC13_ is the amount of the C13 internal standard added at the beginning of the transesterification process, and m is the dry weight of the sample used for transesterification. 

#### 2.5.5. Extraction of Total Pigments and Spectrophotometric Determination of Bacteriochlorophyll-a

The extraction of total pigments synthesized during *R. adriaticum* DSM 2781 cultivation in Erlenmeyer flasks and a bioreactor was performed using a mixture of organic solvents with mechanical cell disruption using glass beads. The sample volume used for pigment extraction was 5 mL for cultivations in Erlenmeyer flasks and 20 mL for those in the bioreactor. The centrifugation of samples was performed at 8000 rpm for 10 min. The supernatant was decanted, and the remaining biomass was used for the extraction and determination of bacteriochlorophyll-a.

To the biomass, 4 g of glass beads (r = 0.5 mm) and 4 mL of an acetone–ethanol mixture (7:2 ratio, vol vol^−1^) were added. The biomass and beads were alternately subjected to intensive mixing for 2 min and cooling for 2 min, repeated three times. To prevent the degradation of photosensitive pigments due to light exposure, the extraction procedure was carried out in the dark, with cuvettes shielded in aluminum foil. After extraction, the sample was centrifuged at 8000 rpm for 10 min.

Spectrophotometric determination (Cary 100 UV-Vis spectrophotometer; Agilent Technologies, Santa Clara, CA, USA) of total pigments in the supernatant after extraction was performed in a quartz cuvette (Hellma Optik GmbH, Jena, Germany) by scanning across the wavelength range of 350 to 900 nm. The concentration of total pigments (expressed as bacteriochlorophyll-a) was calculated using the extraction equation with the ethanol–acetone mixture (7:2 ratio; vol vol^−1^) [58].

(3) $$P=0.348209\times \left(A_{648}-A_{850}\right)-0.16583\times \left(A_{665}-A_{850}\right)+12.11114\times (A_{775}-A_{850})$$

where A represents the value of the absorbance of the sample at a given wavelength.

#### 2.5.6. Statistical Analysis

The standard procedure in the Statistica 12.0 software (StatSoft, Tulsa, OK, USA) was used for calculation of the standard deviations of the experimental data. Standard deviations are presented as bars in tables and figures. In this study, all experiments were repeated at least in duplicate, and the obtained results are shown as average values in tables and figures.

#### 2.5.7. Calculation of Bioprocess Efficiency Parameters

For the calculation of bioprocess efficiency parameters, the following equations were used [59,60]:

(4) $$Y_{S}=C_{S0}-C_{S}$$

(5) $$Y_{P}=C_{P}-C_{P0}$$

(6) $$Y_{P/S}=\frac{Y_{P}}{Y_{S}}$$

(7) $$E=\frac{Y_{P/S}}{Y_{P/ST}}\cdot 100$$

(8) $$Pr=\frac{Y_{P}}{t}$$

*ln C_S_* = *ln C_S_*_0_ + *r_S_*·*t* (9)

*ln X* = *ln X*_0_ + *µ*·*t* (10)

*ln C_P_* = *ln C_P_*_0_ + *r_P_*·*t* (11)

where *C_S_*_0_ and *C_S_* are the initial and final substrate concentrations (g L^−1^), *X*_0_ and *X* are the initial and final biomass concentrations (g L^−1^), *C_PO_* and *C_P_* are the initial and final product concentrations (g L^−1^), *Y_S_* is the total consumption of substrates (g L^−1^), *Y_P_* is the total product yield (g L^−1^), *Y_P/S_* is the conversion coefficient of substrate into product (g g^−1^), *Y_P/ST_* is the theoretical conversion coefficient of substrate into product (g g^−1^), *E* is the bioprocess efficiency (%), *Pr* is the bioprocess productivity (g L^−1^ h^−1^), and *t* is the time (h). The substrate consumption rate (*r_S_*; h^–1^), the specific growth rate (*μ*; h^–1^), and the product synthesis rate (*r_P_*; h^–1^) were determined as first-order reactions using data from exponential phases, and *r_S_*, *μ*, and *r_P_* were determined as the slopes of linearized regression lines according to Doran [60].

## 3. Results and Discussion

### 3.1. Cultivations of Mucor Indicus DSM 2185 

In our preliminary research, the optimization of mixed grass hydrolysis in a high-pressure reactor was conducted. The main objective was to obtain a liquid phase of grass hydrolysates rich in sugars (e.g., glucose and xylose) for use in microbial cultivations. It is worth noting that the liquid phase of lignocellulosic biomass hydrolysates is typically discarded or burned for energy production [61]. The solid fraction of grass hydrolysates needs further enzymatic treatment with enzyme cocktails to hydrolyze the residual cellulose into glucose, although this was not the focus of our research.

#### 3.1.1. Cultivations of *Mucor indicus* DSM 2185 on Media Containing Various Sugars Derived from Grass Hydrolysates 

**Figure 1:**
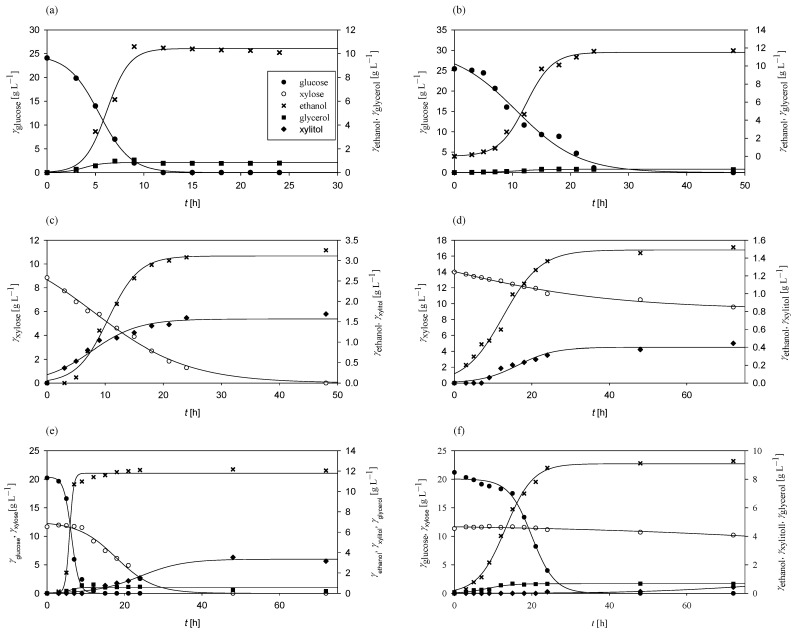
Substrates consumption of and products formation during cultivation of *M. indicus* in Erlenmeyer flasks at 30 °C on media with (**a**) glucose under aerobic conditions, (**b**) glucose under anaerobic conditions, (**c**) xylose under aerobic conditions, (**d**) xylose under anaerobic conditions, (**e**) glucose and xylose under aerobic conditions, and (**f**) glucose and xylose under anaerobic conditions.

At the start of the research, *Mucor indicus* DSM 2185 was cultivated in media containing sugars derived from grass hydrolysates, specifically glucose, xylose, or a combination of both, as carbon sources. The primary objective was to determine the kinetic and bioprocess parameters of *M. indicus* as a potential microorganism for biofuel and biochemical production. The results of these experiments are presented in Figure 1.

Under aerobic conditions with glucose as the sole carbon source, *M. indicus* completely consumed all the available glucose within 12 h, achieving a consumption rate of 0.25 h^−1^ (Figure 1a). The glucose-to-ethanol conversion coefficient was 0.42 g g^−1^, corresponding to 82.35% of the theoretical maximum (0.51 g g^−1^). Ethanol productivity reached 0.42 g L^−1^ h^−1^. Glycerol was also produced as a byproduct, with concentrations ranging from 0.12 to 1.16 g L^−1^. In contrast, under anaerobic conditions (Figure 1b), the glucose consumption rate decreased to 0.10 h^−1^, with complete glucose depletion requiring more than 24 h. The glucose-to-ethanol conversion coefficient increased to 0.46 g g^−1^, resulting in an overall ethanol productivity of 0.24 g L^−1^ h^−1^ due to the extended glucose consumption period. Glycerol was produced under both aerobic and anaerobic conditions.

With xylose as the sole carbon source, *M. indicus* maintained a consumption rate of 0.25 h^−1^ aerobically (Figure 1c), while under anaerobic conditions (Figure 1d) the rate decreased to 0.01 h^−1^. All the xylose was consumed aerobically within 48 h, whereas only 4.42 g L^−1^ was utilized anaerobically over 72 h. The xylose-to-ethanol conversion coefficients were 0.18 g g^−1^ in aerobic conditions and 0.11 g g^−1^ anaerobically, approximately half of the conversion ratio observed with glucose. Bioprocess productivity was consistent, with 0.07 g L^−1^ h^−1^ in aerobic and 0.02 g L^−1^ h^−1^ in anaerobic conditions.

In media containing both glucose and xylose, *M. indicus* first consumed all the glucose within 15 h aerobically (rate of 0.53 h^−1^; Figure 1e) and within 48 h anaerobically (rate of 0.03 h^−1^; Figure 1f). Xylose consumption began only after glucose depletion, indicating diauxic growth. Aerobically, xylose was undetectable after 35 h post-glucose depletion, with a consumption rate of 0.11 h^−1^. Under anaerobic conditions, there was minimal change in xylose concentration, even after 48 h. Byproducts included glycerol and xylitol, with higher xylitol concentrations (3.16 g L^−1^) under aerobic conditions and a lower concentration (0.44 g L^−1^) anaerobically.

Across all experiments (Figure 1), *M. indicus* showed high substrate-to-biomass conversion coefficients, particularly in media with a mix of glucose and xylose, where the coefficient reached 0.29 g g^−1^. The lowest substrate-to-biomass conversion coefficient was observed on xylose (0.01 g g^−1^). These results highlight *M. indicus* as a promising microorganism for biofuel production due to its ability to metabolize both glucose and xylose, tolerate certain inhibitory compounds, and produce ethanol as well as other biochemicals, such as glycerol, xylitol, chitin, chitosan, and fatty acids [62,63,64].

#### 3.1.2. Cultivations of *Mucor indicus* DSM 2185 in Erlenmeyer Flasks on Media Containing Liquid Phase of Grass Hydrolysates (LGH) 

Growth of *M. indicus* DSM 2185 was also investigated using detoxified LGH media containing glucose, xylose, and various nitrogen sources (CSL and yeast extract). Fungi can naturally utilize different substrates to produce a variety of metabolites for different applications. Therefore, they are of great interest as biocatalysts in biorefineries [45,46]. The main *Mucor* species products are lipids, ethanol, organic acids, chitin/chitosan, pigments, and enzymes that can be recovered from the fermentation broth and used in nutrition, chemical, and biofuel industries. In addition, *Mucor* biomass can be directly utilized for specific applications, such as animal feed. Fungi from the *Mucoromycota* phylum can also be used for the development of bioprocesses with simultaneous formation of multiple intra- and extracellular products within a one-step bioprocess. Therefore, the economic viability of the bioprocess can be enhanced [38,46].

**Figure 2:**
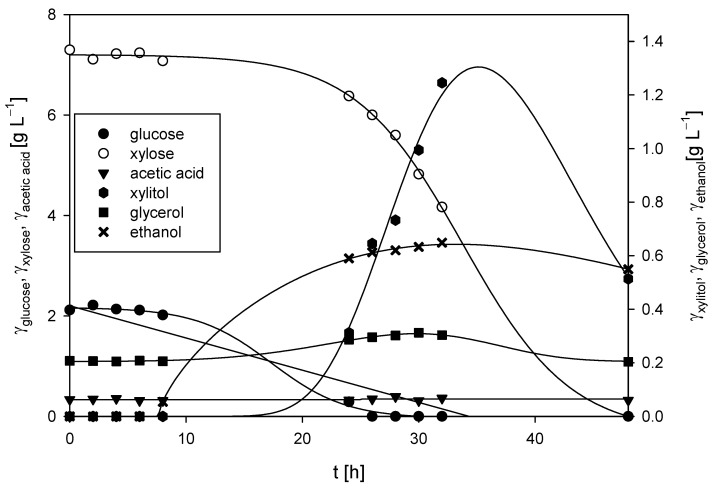
Substrates consumption of and products formation during cultivation of *M. indicus* DSM 2185 in Erlenmeyer flasks on LGH medium without nitrogen source addition.

In our study, the first set of experiments were cultivations of *M. indicus* DSM 2185 on LGH media without the addition of a nitrogen source (Figure 2). After 24 h of cultivation, the fungus depleted all the glucose present in the medium (*∆γ_glucose_* = 2.11 g L^−1^) and then began consuming xylose, showing sequential substrate consumption kinetics. By the end of the process, all of the xylose present in the medium was depleted (∆γ_xylose_ = 7.30 g L^−1^). From the obtained data, glucose and xylose consumption rates of 0.097 h^−1^ and 0.16 h^−1^ were determined, respectively.

The presence of acetic acid in the cultivation media was due to the dilute acid grass hydrolysis process and was in accordance with the literature [2]. Acetic acid present in the medium was not metabolized during the 48 h cultivation period, and its concentration remained nearly constant throughout the fermentation (*γ_acetic acid_* = 0.34 ± 0.03 g L^−1^). After 48 h, 0.55 g L^−1^ of ethanol was produced, resulting in a substrate-to-ethanol conversion coefficient of 0.06 g g^−1^. In addition to ethanol, small amounts of xylitol (*γ_xylitol_* = 0.39 g L^−1^) and glycerol (*γ_glycerol_* = 0.20 g L^−1^) were also produced. The fungal biomass concentration at the end of the bioprocess was 3.09 ± 0.15 g L^−1^.

**Figure 3:**
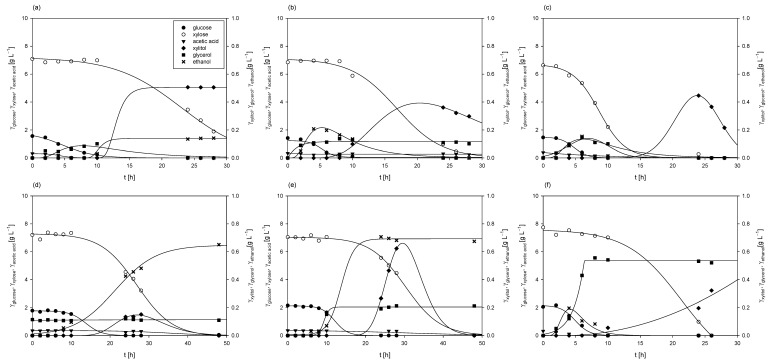
Substrates consumption of and products formation during cultivation of *M. indicus* DSM 2185 in Erlenmeyer flasks on LGH media with addition of different nitrogen sources: (**a**) YE =1 g L^−1^; (**b**) YE =5 g L^−1^; (**c**), YE =10 g L^−1^; (**d**) CSL = 1 g L^−1^; (**e**) CSL = 5 g L^−1^; (**f**) CSL = 10 g L^−1^.

By adding a nitrogen source, either yeast extract or CSL, to the detoxified hydrolysates in all cultivations (Figure 3), after utilizing glucose, the fungus began to use xylose as a carbon source, as was also determined in the cultivation without the addition of a complex nitrogen source. Arabinose, obtained during weak acid pretreatment of grass, was not metabolized, and its concentration remained constant throughout the cultivation processes (3.34 ± 0.14 g L^−1^). It was also detected that the fungus consumed acetic acid within 24 to 48 h of cultivation on both nitrogen sources, which aligns with findings reported in the literature [38,39,65].

Glucose consumption rates were higher with yeast extract (*r_glucose_* = 0.10–0.36 h^−1^) in comparison to cultivations with CSL (*r_glucose_* = 0.06–0.24 h^−1^), while xylose consumption rates were relatively similar (*r_xylose_* = 0.19–0.38 h^−1^). However, it should be noted that the concentrations of biochemicals (ethanol, glycerol, and xylitol) produced were 5 to 10 times lower compared to cultivations on grass hydrolysates without a nitrogen source. In most cases, the fungus assimilated the majority of byproducts by the end of the bioprocesses, as previously reported in the literature [39,40].

Final fungal biomass concentrations were higher compared to cultivations on LGH media without nitrogen source addition, ranging from 4.09 ± 0.21 g L^−1^ to 5.28 ± 0.35 g L^−1^ when using yeast extract and from 3.97 ± 0.14 g L^−1^ to 5.61 ± 0.24 g L^−1^ when CSL was used. The highest biomass concentrations in both cases were achieved when 5 g L^−1^ of the nitrogen source was added to the detoxified LGH, with a slightly higher concentration (6.33% higher) observed when using CSL.

The cell wall of *M. indicus* is rich in chitin and chitosan. Significant amounts of biomass are formed during aerobic processes, and the proportions of chitin and chitosan change as the fungus morphology shifts from individual cell growth to a filamentous form [62]. Chitin and chitosan were isolated from the fungal biomass due to their potential applications in the food, cosmetic, and pharmaceutical industries [62]. 

**Figure 4:**
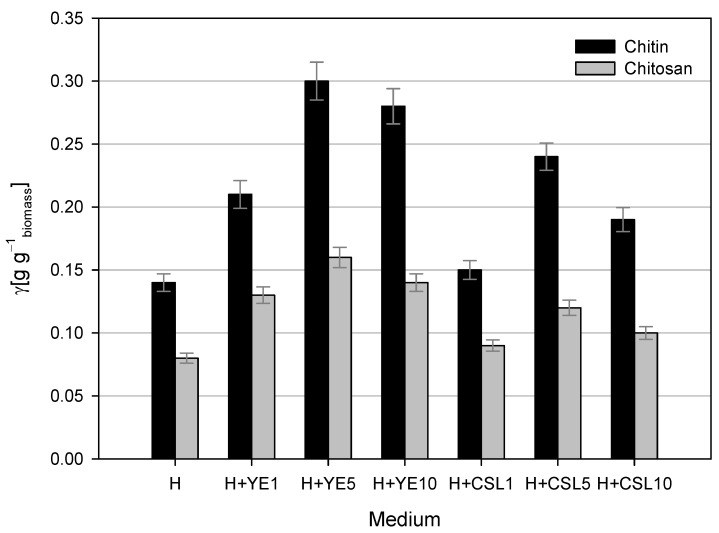
Chitin and chitosan yields from dry fungal biomass after cultivations in Erlenmeyer flasks on LGH media (H) containing various concentrations of yeast extract (YE) and corn steep liquor (CSL).

A comparison of chitin and chitosan yields from the dry fungal biomass grown on media with different nitrogen sources (Figure 4) showed that the yields varied depending on the medium composition. The highest yields of chitin and chitosan were observed after cultivation on LGH media with the addition of 5 g L^−1^ yeast extract. At the end of the cultivation, the dry biomass concentration was 5.28 ± 0.35 g L^−1^, and 0.30 ± 0.0108 g g^−1^ of chitin and 0.16 ± 0.006 g g^−1^ of chitosan were isolated from the dry biomass. Cultivation in other media with yeast extract resulted in slightly lower yields of chitin (0.21–0.28 g g^−1^) and chitosan (0.13–0.14 g g^−1^). The biomass obtained after cultivation on LGH media with CSL as the nitrogen source contained less chitin and chitosan compared to that obtained from cultivation with yeast extract. LGH media with 1 g L^−1^ CSL resulted in half the yield of chitin and chitosan compared to the cultivation with 5 g L^−1^ yeast extract. The highest yields of chitin and chitosan in this set of experiments were achieved using 5 g L^−1^ CSL, though they were slightly lower than the maximum values (0.24 ± 0.108 g g^−1^ of chitin and 0.12 ± 0.006 g g^−1^ of chitosan). Dry biomass obtained after cultivation with 10 g L^−1^ CSL contained 37% less chitin and 56.25% less chitosan than the maximum yields. The lowest yields of chitin and chitosan from dry biomass of *M. indicus* were obtained after cultivation in pure LGH medium, showing reductions of 54% and 50%, respectively, compared to the maximum values.

In comparison, Sharifyazd and Karimi [63] determined the yield of chitin and chitosan in dry biomass to be 0.21 g g^−1^ and 0.16 g g^−1^, respectively. Based on their results, the yield of chitin obtained after the cultivation of *M. indicus* on LGH medium with the addition of 5 g L^−1^ yeast extract was approximately 40% higher, while the yield of chitosan remained at approximately the same level. In addition to aeration, the chitin and chitosan contents in the dry biomass of *M. indicus* were also affected by the cultivation temperature, with the highest chitin content observed at 28 °C and the content decreasing as the temperature increased, unlike chitosan, whose content was lowest at 28 °C and increased with rising temperature [63].

Many eukaryotic microorganisms store energy in cells as triacylglycerols and lipids, which are integral components of membranes, in the form of phospholipids. High amounts of lipids in the form of triacylglycerides (TAGs) with fatty acid profiles similar to plant and fish oils are obtained by cultivation of oleaginous filamentous fungi under nitrogen-limitation conditions [66]. *M. indicus* produces fatty acids using acetyl-CoA and utilizes dihydroxyacetone phosphate for the synthesis of triacylglycerols and phospholipids [66].

Gas chromatography analysis of the fatty acid methyl ester (FAME) compositions, obtained after transesterification and extraction from the fungal biomass samples, detected 22 different fatty acids present in varying concentrations (see Table 2). The most abundant fatty acids were palmitic acid (C16:0), linoleic acid (C18:2 trans 9,12), linolenic acid (C18:2 cis 9,12), and *γ*-linolenic acid (C18:3), with oleic acid (C18:1 cis 9) comprising from 15.4% to nearly 50% of the total fatty acids in the dry biomass, depending on the composition of the medium used.

The total fatty acid content in the dry biomass obtained after cultivation on LGH medium without a nitrogen source was 6.08%. With the addition of yeast extract, the fatty acid content in the biomass ranged from 5.66% at the lowest yeast extract concentration to 4.18% at 10 g L^−1^ yeast extract. In contrast, after cultivation with 1 g L^−1^, 5 g L^−1^, and 10 g L^−1^ CSL, the biomass contained 7.20%, 15.76%, and 15.25% fatty acids, respectively (Table 2). The addition of yeast extract as a nitrogen source did not significantly affect the total fatty acid content and even caused a slight decrease, while media containing CSL as the nitrogen source showed a 1.18-to-2.51-fold increase in total fatty acid contents. Cultivation with CSL proved to be more successful in terms of both total fatty acid mass and fatty acid content in the biomass. The highest fatty acid content in this set of experiments, 15.76%, was obtained after analyzing the biomass cultivated in LGH medium with 5 g L^−1^ CSL. This resulted in 120.80 ± 3.75 mg g^−1^ of fatty acids, which was 141.70% more than the 50.43 ± 0.86 mg g^−1^ produced during cultivation on LGH medium without a nitrogen source and 120.10% more than on the same medium with 5 g L^−1^ yeast extract.

The influence of nitrogen source concentration on lipid production aligns with data available in the literature, specifically with the studies of Sharifyzad and Karimi [63] and Dzurendova et al. [66], where they noted that lower nitrogen concentrations result in higher lipid yields. One strategy for inducing intracellular lipid synthesis is nitrogen limitation. Under these conditions, microorganisms rapidly assimilate all available nitrogen and continue to assimilate the available carbon source, storing it in cells as lipids [65]. Shafiei Alavijeh et al. [62] cultivated *M. indicus* using corn stover hydrolysates enriched with yeast extract and ammonium sulfate as nitrogen sources. In their experiments, *M. indicus* produced 147.4 ± 2 mg of lipids per gram of assimilated sugar from the substrate, equal to 19.3% of the biomass dry weight. This result is slightly higher compared to the maximum total fatty acid content obtained in this study using CSL as the nitrogen source but almost 3.5 times higher than the total fatty acid content of the dry biomass obtained with yeast extract. While the most abundant fatty acids in the biomass in this study were oleic, linoleic, and *γ*-linolenic acids, as previously mentioned, in the study by Shafiei Alavijeh et al. [62], stearic acid (53.5% of all fatty acids) was predominantly produced, along with palmitoleic (22.7%) and palmitic acid (13.7%).

### 3.2. Cultivations of Bacterium Rhodovulum adriaticum DSM 2781 in Erlenmeyer Flasks

**Figure 5:**
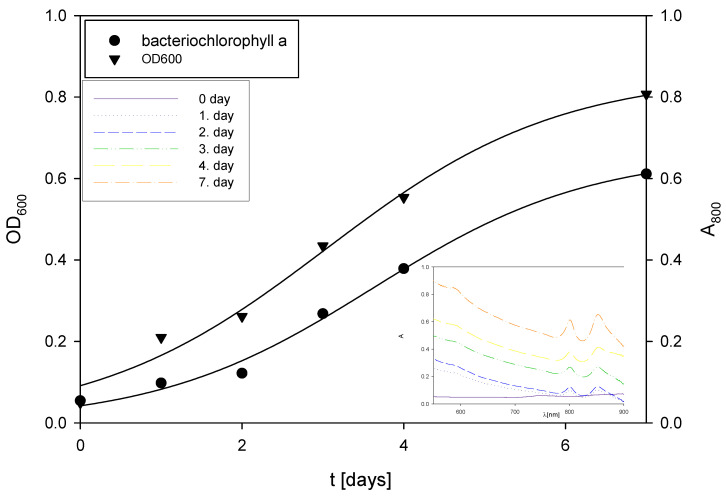
Changes in OD_600_ and absorbance during the cultivation of *R. adriaticum* in Erlenmeyer flasks on exhausted LGH medium without the addition of a nitrogen source.

**Figure 6:**
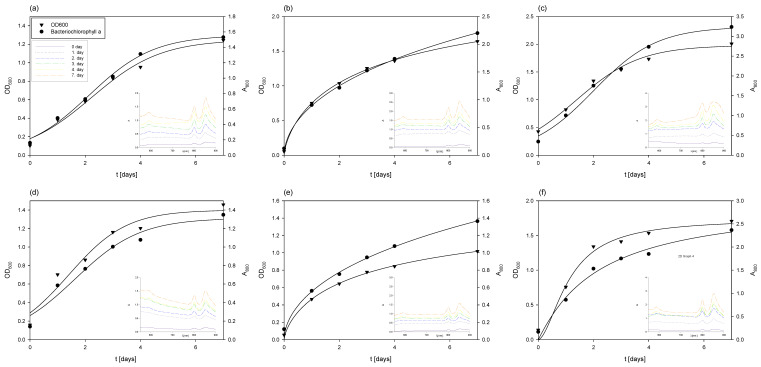
Changes in OD_600_ and absorbance during the cultivation of *Rhodovulum adriaticum* in Erlenmeyer flasks on exhausted LGH media which previously contained the following: (**a**) 1 g L^−1^ yeast extract (YE); (**b**) 5 g l^−1^ YE; (**c**) 10 g L^−1^ YE; (**d**) 1 g L^−1^ CSL; (**e**) 5 g L^−1^ CSL; (**f**) 10 g L^−1^ CSL.

The bacterium *R. adriaticum* DSM 2781 was cultivated under microaerophilic phototrophic conditions on residual media obtained after the cultivation of *M. indicus* DSM 2185 in LGH media with varying concentrations of CSL and yeast extract as nitrogen sources (Figure 5 and Figure 6). It has already been shown that purple non-sulfur bacteria can grow on alcohols, organic acids, and similar metabolic byproducts of other microorganisms [41,42,43,44]. In this study, the potential growth of *R. adriaticum* and the production of valuable products such as pigments from the remaining nutrients in the medium after the cultivation of *M. indicus* were examined. The media contained low concentrations of ethanol, acetic acid, glycerol, and xylitol. By utilizing spent media for the cultivation of bacterial biomass and the production of valuable products, an even higher level of lignocellulosic raw material utilization could be achieved.

During the first two days of cultivation on the exhausted LGH medium without nitrogen source addition (Figure 5), *R. adriaticum* DSM 2781 began assimilating ethanol, acetic acid, and xylitol present in the medium. Bacterial growth and total pigment concentrations were monitored spectrophotometrically, and the results are shown in Figure 5. Concentrations of bacteriochlorophyll-a followed the biomass growth curve, with a steady increase in both biomass and bacteriochlorophyll-a concentrations.

The final bacterial biomass concentration was determined gravimetrically and found to be 0.57 g L^−1^. This concentration is lower than the values reported in the literature, which was to be expected due to the low initial concentrations of carbon sources in the medium. At the end of the cultivation, 1.02 ± 0.09 g L^−1^ of isopropanol was present in the medium, which could be considered a valuable product alongside pigments or the bacterial biomass itself. Isopropanol can be produced through the action of transferase on acetoacetyl-CoA, that is, acetoacetate is formed, which then undergoes decarboxylation to produce acetone and CO₂. Subsequently, alcohol dehydrogenase can convert the resulting acetone into isopropanol. This synthetic pathway is a hypothesis, given that this bacterium can assimilate isopropanol as a carbon source [67,68]. At the end of the bioprocess, total pigments were extracted from the bacterial biomass, and the concentration of bacteriochlorophyll-a was measured. The concentration of bacteriochlorophyll-a in the bacterial biomass at the end of the cultivation was 6.84 ± 0.12 mg g^−1^.

In the case of cultivation on the exhausted LGH medium (Figure 6), which initially contained 1 g L^−1^ of CSL during the cultivation of *M. indicus*, the concentration of *R. adriaticum* biomass at the end of cultivation was 0.46 ± 0.01 g L^−1^. This was 1.24 times lower compared to the biomass concentration at the end of cultivation on the exhausted LGH medium without nitrogen source addition. A possible reason is that the bacteria did not assimilate the available substrates remaining from the previous cultivation (ethanol, glycerol, acetic acid, and xylitol). However, although less bacterial biomass was produced on this medium, the biomass at the end of the bioprocess contained three times more bacteriochlorophyll-a, specifically 18.19 ± 0.21 mg g^−1^.

The cultivation of *R. adriaticum* using the exhausted LGH medium that initially contained 5 g L^−1^ CSL proved to be more effective in terms of biomass production. The optical density increased steadily during cultivation, reaching its maximum value on the 7th day. This was further confirmed at the end of the cultivation, when the bacterial biomass concentration, determined gravimetrically, was 0.71 ± 0.06 g L^−1^. Moreover, this cultivation also demonstrated higher efficiency in bacteriochlorophyll-a production. After extraction, the spectrophotometrically determined concentration was 25.2 ± 0.66 mg g^−1^.

During cultivation on the exhausted LGH medium, which initially contained 10 g L^−1^ of CSL, there was a slight increase in biomass concentration after 7 days of cultivation, reaching 0.8 ± 0.03 g L^−1^. Interestingly, a lower initial concentration of CSL (5 g L^−1^) proved to be more suitable for bacteriochlorophyll-a production, with 25.2 ± 0.96 mg g^−1^ of bacteriochlorophyll-a detected at the end of this cultivation. This was higher compared to the cultivation on the exhausted LGH medium that initially contained 10 g L^−1^ of CSL, which yielded 15.15 ± 0.62 mg g^−1^. 

During the 7-day cultivation of *R. adriaticum* on the exhausted LGH medium that initially contained 1 g L^−1^ of yeast extract, only minimal changes in ethanol concentration were observed. However, after the second and third day of cultivation, there was a sharp decrease in acetic acid and glycerol concentrations. It is likely that the bacteria utilized acetic acid and ethanol for growth, as the biomass concentration at the end of cultivation, determined gravimetrically, was 0.72 ± 0.03 g L^−1^—representing a 56% increase compared to the biomass concentration obtained on the exhausted LGH medium with the same CSL concentration. Additionally, the concentration of bacteriochlorophyll-a measured at the end of cultivation on the LGH medium with initially 1 g L^−1^ of yeast extract was 33.04 ± 1.21 mg g^−1^.

In terms of bacteriochlorophyll-a production, the highest yield was observed in the cultivation on the exhausted LGH medium, with an initial yeast extract concentration of 5 g L^−1^, where 48.1 ± 0.96 mg g^−1^ of bacteriochlorophyll-a was determined. The rapid decrease in ethanol, xylitol, and acetic acid concentrations after the first day of cultivation suggested that the bacterium utilized these substrates for growth, which was also reflected in the increase in bacterial biomass concentration. The final bacterial biomass concentration at the end of cultivation was 0.98 ± 0.11 g L^−1^.

Cultivation of *R. adriaticum* DSM 2781 on the exhausted LGH medium, which initially contained 10 g L^−1^ of yeast extract, resulted in the highest production of bacterial biomass. At the end of cultivation, the biomass concentration was 1.85 ± 0.042 g L^−1^, representing a 132% increase in biomass yield compared to the next best cultivation, which used the exhausted LGH medium with an initial concentration of 10 g L^−1^ CSL, where 0.80 ± 0.05 g L^−1^ of biomass was obtained. Regarding bacteriochlorophyll-a production, this cultivation performed better than those conducted in media previously containing CSL, but among the yeast extract cultivations, the LGH medium with initially 10 g L^−1^ had the lowest pigment production. After extraction, 20.8 ± 1.28 mg g^−1^ of bacteriochlorophyll-a was determined at the end of cultivation.

In the study by Novak et al. [42], the authors reported on biomass and pigment production by *Rhodovulum adriaticum* DSM 2781 cultivated on media containing glucose and xylose as carbon sources. By cultivating the bacterium on 3 g L^−1^ of glucose and xylose, a productivity of 0.017 g L^−1^ h^−1^ was achieved, with a final biomass yield of 1.507 g L^−1^. Since glucose and xylose are the predominant carbohydrates in hydrolysates of lignocellulosic feedstock, these results can be compared to the cultivation of *R. adriaticum* in the present study. As previously mentioned, the highest bacterial biomass concentration in this study was achieved at the end of cultivation on detoxified LGH medium that initially contained yeast extract, reaching 1.85 ± 0.29 g L^−1^ with a productivity of 0.011 ± 0.01 g L^−1^ h^−1^. The lower biomass yield compared to Novak et al. [42] could be attributed to the significantly lower substrate concentrations remaining in the detoxified LGH medium after the cultivation of *M. indicus*, as well as slightly different cultivation conditions. Purple non-sulfur bacteria can grow under a variety of metabolic conditions, including photoautotrophic, chemoautotrophic, photoheterotrophic, and chemoheterotrophic modes, utilizing different elements as electron acceptors. They are also capable of both fermentation and cellular respiration. Their ability to maintain respiratory activity even under photoautotrophic growth conditions allows these bacteria to adapt their metabolism effectively to environmental changes. Under photoheterotrophic conditions, when carbon and nitrogen sources are abundant, the synthesis of photosynthetic pigments may be repressed [41,42,43,44,45]. This is consistent with our results, which show higher biomass concentrations accompanied by lower pigment concentrations.

### 3.3. Cultivations of Mucor indicus DSM 2185 and Rhodovulum Adriaticum in Bioreactors

#### 3.3.1. Cultivation of *M. indicus* DSM 2185 in a Bubble Column Bioreactor

**Figure 7:**
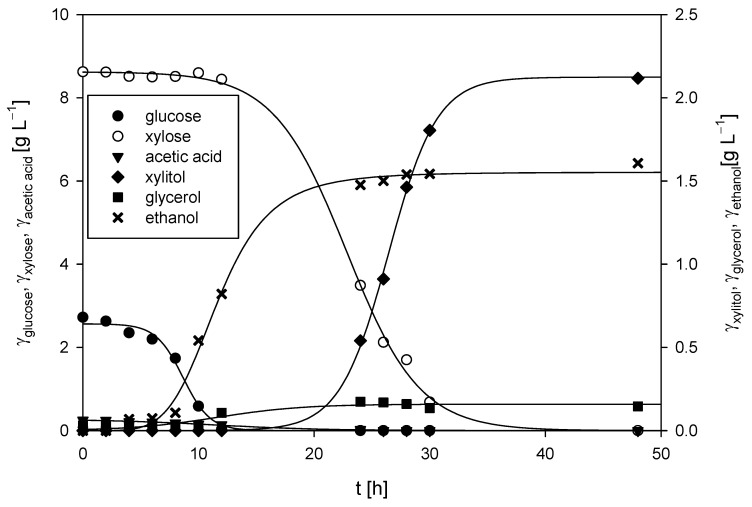
Changes in concentrations of substrates and products during *M. indicus* DSM 2185 cultivation on LGH media with the addition of a nitrogen source in a bubble column bioreactor.

The cultivation of *M. indicus* DSM 2185 in a bubble column bioreactor with a working volume of 1.5 L was conducted aerobically, with an airflow rate of 2 L h^−1^. The LGH medium was supplemented with 5 g L^−1^ of CSL, as this concentration of the nitrogen source resulted in the highest fatty acid content and some of the best chitin and chitosan yields from the fungal biomass. The time required for the fungus to consume all the available carbon sources, both glucose and xylose, was 48 h (Figure 7). During the first 12 h of cultivation, the glucose concentration decreased from the initial 2.72 ± 0.13 g L^−1^ to 0.03 g L^−1^ (glucose was undetectable after 24 h), while the xylose concentration remained almost unchanged (≈8.6 ± 0.18 g L^−1^). During the first 12 h, the ethanol concentration increased to 0.82 ± 0.09 g L^−1^, which resulted in a productivity of 0.068 ± 0.01 g L^−1^ h^−1^. After 12 h, a continuous decrease in the xylose concentration was observed, with complete depletion of xylose by 48 h. During the remaining 36 h, the ethanol concentration increased from 0.82 ± 0.06 g L^−1^ to a final concentration of 1.61 ± 0.10 g L^−1^, resulting in a slightly lower productivity of 0.022 ± 0.01 g L^−1^ h^−1^ when the fungus predominantly utilized xylose compared to glucose.

Glycerol was detected in the medium only after 12 h, reaching a maximum concentration of 0.17 g L^−1^ after 24 h. The concentration of xylitol correlated with xylose consumption, as xylitol was first detected at 10 h, and its concentration increased steadily to a maximum of 2.12 ± 0.11 g L^−1^ by the end of cultivation. The fungus did not utilize arabinose, and its concentration remained constant throughout the process (3.36 ± 0.12 g L^−1^). The final biomass concentration of 5.91 ± 0.08 g L^−1^ was slightly higher than that achieved on the same LGH medium in Erlenmeyer flasks, where the final biomass concentration was 5.61 ± 0.24 g L^−1^.

Sues et al. [69] conducted the cultivation of *M. indicus* in a bioreactor of the same volume (1.5 L) using spruce forest waste hydrolysates. Their hydrolysates contained significantly more sugars—specifically, 52.5 g L^−1^—compared to the hydrolysates used in this study, which contained 11.32 g L^−1^ of sugars. Consequently, the ethanol yield in their cultivation was higher, amounting to 0.18 g g^−1^, while the sugar-to-ethanol conversion coefficient at the end of the cultivation in this study was 0.14 g g^−1^ (Table 3).

There are additional examples in the literature of ethanol production using *M. indicus* on hydrolysates derived from lignocellulosic raw materials. Satari et al. [64] successfully produced ethanol under aerobic conditions from hydrolysates of rice straw, mushroom compost, and pine, achieving sugar-to-ethanol conversion coefficients of 0.37 g g^−1^, 0.33 g g^−1^, and 0.25 g g^−1^, respectively. Lennartsson et al. [70] cultivated *M. indicus* on hydrolysates derived from orange peels. Under aerobic conditions, the fungus produced 0.41 g of ethanol per gram of hexoses and 57 mg g^−1^ of biomass in Erlenmeyer flasks, while in a bioreactor, the hexose-to-ethanol conversion coefficient was 0.40 g g^−1^, with a substrate-to-biomass conversion coefficient of 0.75 mg g^−1^.

Additionally, Sues et al. [69] conducted cultivations on hydrolysates of forest wood waste, primarily composed of spruce residues, and produced 0.42 g of ethanol per gram of assimilated sugars from the nutrient medium. 

After the cultivation in the bubble column bioreactor, a slightly higher yield of chitin and chitosan was achieved (Table 3) compared to the flask cultivations. The chitin yield was 0.31 ± 0.015 g g^−1^, while 0.17 ± 0.0062 g g^−1^ of chitosan was isolated from the dry biomass. These yields were nearly identical to those obtained from the biomass after flask cultivations using LGH medium enriched with 5 g L^−1^ of yeast extract.

Although a higher fatty acid content in the biomass was anticipated, this cultivation proved less successful compared to similar experiments conducted in Erlenmeyer flasks. This occurrence shows that further optimization of *M. indicus* cultivation in the bubble column bioreactor has to be performed in order to improve bioprocess performance and efficiency. During the 48 h bioreactor cultivation, only 0.41 ± 0.03 g of total fatty acids were produced, constituting 6.94% of the biomass—nearly 2.5 times lower than the fatty acid content observed in the fungal biomass from the Erlenmeyer flask cultivations using the same medium. Out of the 22 fatty acid methyl esters previously detected, only 12 were found in the biomass obtained from the stirred-tank bioreactor. These included hexanoic acid (C6:0), caprylic acid (C8:0), undecanoic acid (C11:0), dodecanoic acid (C12:0), myristic acid (C14:0), palmitic acid (C16:0), palmitoleic acid (C16:1 cis 9), stearic acid (C18:0), oleic acid (C18:1 cis 9), linolelaidic acid (C18:2 trans 9,12), linoleic acid (C18:2 cis 9,12), and *γ*-linolenic acid (C18:3 cis 6,9,12).

The most often present fatty acids in this study were palmitic acid (C16:0; 6.69 ± 0.10 mg g^−1^), oleic acid (C18:1; 17.41 ± 0.16 mg g^−1^), linolelaidic acid (C18:2 trans 9,12; 4.83 ± 0.33 mg g^−1^), linoleic acid (C18:2 cis 9,12; 6.24 ± 0.29 mg g^−1^), and *γ*-linolenic acid (C18:3; 6.50 ± 0.31 mg g^−1^), while the concentrations of all the other fatty acids were below 1.5 mg g^−1^. When Shafieri Alavijeh et al. [62] conducted similar experiments with *M. indicus* on corn stover hydrolysates, the fatty acid profile differed slightly from that observed in our study. A potential reason for the lower fatty acid content in this study is that the cultivation in the stirred-tank bioreactor was conducted under aerobic conditions. Satari et al. [64] noted that during anaerobic cultivation there is a partial accumulation of carbon dioxide, which is necessary for fatty acid synthesis. Although the flask cultivations were not strictly anaerobic but rather microaerobic, it was assumed that CO_2_ levels were higher during those cultivations, contributing to increased fatty acid production.

Additionally, the growth morphology of *M. indicus* could have influenced the fatty acid composition. Satari et al. [64] concluded that when the fungus grows as individual colonies, it accumulates more fatty acids compared to filamentous growths. This may explain the lower fatty acid content in the biomass from the bubble column bioreactor, as the fungus predominantly grew in a filamentous form, potentially hindering fatty acid accumulation.

#### 3.3.2. Cultivation of *R. adriaticum* DSM 2781 in a Stirred-Tank Bioreactor

**Figure 8:**
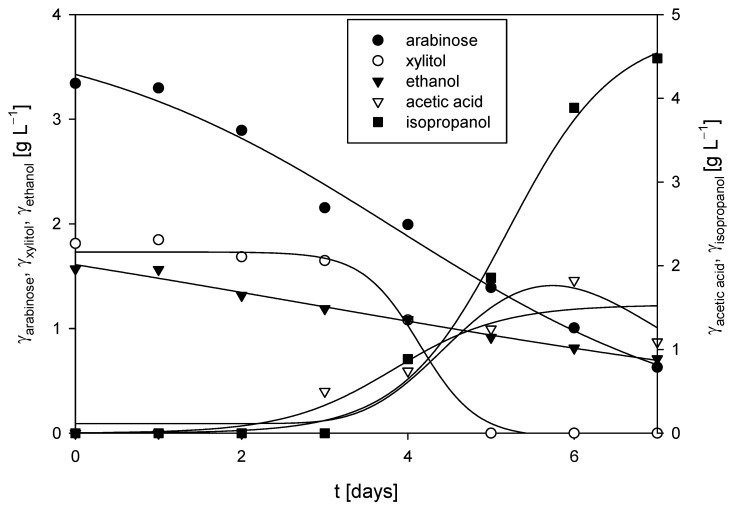
Substrates consumption of and products formation during cultivation of *R. adriaticum* DSM 2781 in a stirred-tank bioreactor.

The cultivation of *R. adriaticum* DSM 2781 was carried out in a stirred-tank bioreactor under anaerobic conditions, utilizing the spent medium from the cultivation of *M. indicus* DSM 2185 on detoxified LGH, with 5 g L^−1^ of CSL as the nitrogen source. No glucose or xylose was detected in the medium. At the start of the process, 3.34 ± 0.14 g L^−1^ of arabinose was present as a carbon source, and its concentration gradually decreased, reaching 0.63 ± 0.06 g L^−1^ by the end of cultivation, suggesting that the bacteria likely utilized arabinose for growth. Additionally, xylitol appeared to be assimilated, as its concentration dropped from 1.81 ± 0.10 g L^−1^ after the fourth day of cultivation. A significant increase in acetic acid and isopropanol concentrations was detected after the third day of cultivation, with isopropanol reaching 4.48±0.68 g L^−1^ at the end of the process (Figure 8). Under nitrogen-sufficient conditions, *R. adriaticum* can shift from producing polyhydroxybutyrate to isopropanol via acetoacetyl-CoA. The process productivity for acetic acid and isopropanol production was 0.01 g L^−1^ h^−1^ and 0.03 g L^−1^ h^−1^, respectively. At the conclusion of the cultivation in the bioreactor, the biomass concentration, measured gravimetrically, was 2.26 ± 0.07 g L^−1^, which was the highest biomass concentration recorded in this study. However, the concentration of bacteriochlorophyll-a was relatively low at 1.78 ± 0.12 mg L^−1^.

Comparing these results to the study by Novak et al. [42], the biomass productivity was 23.5% lower in this study. Their medium contained 3 g L^−1^ each of glucose and xylose, and the cultivation was performed under aerobic conditions, likely accounting for the higher biomass yield in their experiments. However, in this study, the concentration of bacteriochlorophyll-a was notably higher than in Novak et al.’s results [42]. Additionally, they did not report the production of isopropanol or acetic acid, suggesting differences in metabolic pathways under the respective cultivation conditions. 

## 4. Conclusions

Fungi are microorganisms with significant potential for the production of various valuable bioproducts. One notable representative of fungi is *Mucor indicus*, whose metabolism is capable of synthesizing diverse metabolic products, including biofuels and other valuable chemicals. In this study, *M. indicus* DSM 2185 was selected for the production of ethanol, chitin, chitosan, and fatty acids. It was cultivated in media containing the liquid phase of grass hydrolysates (LGH) and different nitrogen sources (YE and CSL). The results clearly demonstrate that bioprocess efficiency is highly dependent on the composition of the cultivation media, as well as the cultivation methods and conditions. The highest bioprocess efficiency parameters were observed during the cultivation of *M. indicus* on LGH media supplemented with CSL. Under these conditions, the highest fungal biomass concentrations were achieved, along with the highest total lipid content in dry biomass (up to 15.76%). Oleic acid (approximately 50%) was the predominant fatty acid in the fungal lipids. Fungal biomass also serves as a valuable source of other chemicals, such as chitin and chitosan, with yields ranging from 0.1 g g^−1^ to 0.3 g g^−1^ dry biomass, depending on the cultivation media and conditions. 

Within a biorefinery framework, the residual medium after *M. indicus* cultivation can be used as a broth constituent for the growth of other microorganisms. For this purpose, the non-sulfur purple bacterium *Rhodovulum adriaticum* DSM 2781 was cultivated on residual LGH broth to produce pigments, primarily bacteriochlorophyll-a, which has a broad range of applications. The results indicate that *R. adriaticum* grew relatively well on the residual LGH medium, with the highest bacteriochlorophyll-a concentration observed in medium supplemented with yeast extract. These findings highlight the significant potential of the established biorefinery system. However, further optimization and improvement are necessary to meet the requirements for successful scale-up and industrial application.

## Figures and Tables

**Table 1:** Parameters of the GC-FID method for determining the composition and concentration of fatty acid esters, including column type, carrier gas, injection settings, oven temperature program, and FID settings.

Parameters	Conditions
Column	ZB-FAME (Zebron, Newport Beach, CA, USA), 30 m × 0.25 mm, df 0.20 μm
Detector	FID
Carrier gas/flow	Helium/1.2 mL min^−1^
Temperature program	10 °C/min → 140 °C 3 °C/min → 190 °C 30 °C/min do 260 °C 260 °C, 2 min
Injector temperature	250 °C
Detector temperature	260 °C
Partition coefficient	1:15
Injection volume	2 μL

**Table 2:** Compositions and yields of fatty acids obtained from fungal biomass cultivated in Erlenmeyer flasks on LGH media (H) with yeast extract (YE) and CSL as nitrogen sources.

Medium	H	H YE1	H YE5	H YE10	H CSL1	H CSL5	H CSL10
FAME	FAME [mg g_biomass_^−1^]						
C6:0	1.18 ± 0.09	1.09 ± 0.01	0.95 ± 0.02	0.91 ± 0.01	0.93 ± 0.01	1.01 ± 0.06	0.97 ± 0.01
C11:0	0.82 ± 0.07	0	0	1.96 ± 0.09	0.63 ± 0.01	1.20 ± 0.08	1.16 ± 0.09
C12:0	0.23 ± 0.03	0	0.33 ± 0.01	0.21 ± 0.01	0.18 ± 0.01	0.18 ± 0.01	0.17 ± 0.01
C14:0	0.24 ± 0.04	0.35 ± 0.1	0.90 ± 0.01	1.58 ± 0.06	0.52 ± 0.01	0.53 ± 0.01	0.51 ± 0.01
C14:1 cis 9	0	1.02 ± 0.01	1.30 ± 0.05	1.50 ± 0.04	0.15 ±0.01	0	0
C15:0	0.05 ± 0.01	0.18 ± 0.01	0.16 ± 0.01	0.99 ± 0.01	0.29 ± 0.01	0.05 ± 0.01	0.05 ± 0.01
C15:1 cis 10	0	0.01	0.23 ± 0.01	0	0	0	0
C16:0	7.63 ± 0.65	5.61 ± 0.24	6.36 ± 0.29	9.27 ± 0.03	7.48 ± 0.32	7.09 ± 0.32	6.86 ± 0.25
C16:1 cis 9	0.55 ± 0.01	0.63 ± 0.01	2.24 ± 0.11	3.49 ± 0.10	1.18 ± 0.10	3.65 ± 0.18	3.53 ± 0.30
C17:0	0	0	0	0.47 ± 0.01	0	4.72 ± 0.29	4.56 ± 0.27
C17:1 cis 10	0	0	0	0.60 ± 0.02	0	0	0
C18:1 cis 9	22.81 ± 0.94	19.41 ± 0.75	19.97 ± 0.67	24.17 ± 1.24	20.49 ± 1.20	18.61 ± 0.78	18.01 ± 0.11
C18:2 trans 9,12	6.99 ±	5.14 ± 0.23	6.86 ± 0.53	10.60 ± 0.98	7.18 ± 0.40	5.45 ± 0.31	5.56 ± 0.08
C18:2 cis 9,12	3.12 ±	3.88 ± 0.16	6.20 ± 0.57	8.55 ± 0.65	4.22 ± 0.22	3.08 ± 0.05	2.99 ± 0.12
C18:3 cis 6,9,12	6.82 ±	7.58 ± 0.41	8.71 ± 0.70	10.23 ± 0.78	8.03 ± 0.36	14.19 ± 1.01	13.74 ± 0.79
C20:1 cis 11	0	0	0	0	0	12.71 ± 0.99	12.30 ± 0.91
C20:4 cis 5,8,11,14	0	0	0	0	0	6.89 ± 0.21	6.47 ± 0.36
C20:5 cis 5,8,11,14,17	0	0	0	0	0	0	11.55 ± 0.88
C22:6 cis 4,7,10,13,16,19	0	0	0	0	0	7.65 ± 0.38	7.40 ± 0.55
C23:0	0	0	0	0	0	0.32 ± 0.01	0.31 ± 0.01
C24:0	0	0	0	0	0	12.27 ± 0.79	11.88 ± 0.07
C24:1 cis 15	0	0	0	0	0	9.16 ± 0.80	0
*w* (FAME) in biomass [%]	6.08 ± 0.35	5.66 ± 0.29	4.18 ± 0.17	4.97 ± 0.21	7.20 ± 0.42	15.76 ± 0.98	15.25 ± 0.83

**Table 3:** Bioprocess efficiency parameters of *M. indicus* DSM 2185 cultivation in a bubble column bioreactor.

*γ_X_* [g L^−1^]	*γ_chitin_* [g g^−1^]	*γ_chitosan_* [g g^−1^]	*Y_etanol_* [g L^−1^]	*Y_Et/S_* [g g^−1^]	*Pr* [g L^−1^ h^−1^]	*q_S_* [h^−1^]	*q_P_* [h^−1^]
5.910	0.310	0.170	1.610	0.142	0.034	0.0364	0.0868

## Data Availability

The data presented in this study are available on request from the corresponding author.
